# Spatial Analysis of Global Prevalence of Multiple Sclerosis Suggests Need for an Updated Prevalence Scale

**DOI:** 10.1155/2014/124578

**Published:** 2014-02-16

**Authors:** Brett J. Wade

**Affiliations:** Department of Therapist Assistant, Okanagan College, 1000 KLO Road, Kelowna, BC, Canada V1Y 4X8

## Abstract

Multiple sclerosis (MS) is a demyelinating disease of the central nervous system with an unknown aetiology. MS has a geographic pattern of prevalence with high prevalence rates between 45 degrees and 65 degrees north. In much of the northern hemisphere, there exists a prevalence gradient, with increasing prevalence from south to north. While genetics may partially explain the latitudinal gradient, it is not strong enough to exclude exogenous variables. Kurtzke initially came up with a three-zone scale for low, medium, and high prevalence zones. He defined high as 30 or more per 100,000, medium as 5–29 per 100,000, and low as less than 5 per 100,000. In this study, 131 geographic datasets (geocases) were spatially analyzed to determine whether the existing global prevalence scale needed to be updated. The mean prevalence rate was 67.83/100,000 with rates ranging from 350/100,000 to 0/100,000. The results of this study suggest that the commonly referenced scale for global MS prevalence needs to be updated with added zones to reflect significantly higher prevalence rates in some areas of the world. We suggest a five-zone scale: very high (170–350), high (70–170), medium (38–70), low (13–38), and very low (0–13).

## 1. Introduction

MS is most common in people of northern European ancestry. It has been concluded by many that genetics are an important factor in disease expression; however, genetics alone are not enough to guarantee the development of MS. Monozygotic twin studies indicate a clinical concordance rate between twenty and thirty percent [1], compared with a two percent to five percent same-sex fraternal twin rate. The National MS Society states that when a sibling or parent has MS, the chance of developing MS is “1 in 40” [2]. It appears that some environmental factor is required to interact with genetics to trigger the onset of MS. Some environmental agents that have been studied as a possible cofactor in the expression of MS include viruses, hormones, vitamin D deficiency, UVB deficiency, diet, smoking, and others [3]. Studying the effects of environment on disease expression can be perplexing. Factors such as length of time of exposure to different factors, climate, migration, and occupation are all potential confounders.

A genetic link to MS may partially explain the geographic distribution of the disease as Caucasoid races tended to migrate towards the temperate climates [4]. An interesting feature of MS is that it seems to have an age of susceptibility. Migration studies have discovered that age of MS risk is around the age of pubescence [1]. If a person moves from a low-risk area to a high-risk area before pubescence, the person will be susceptible to the level of risk in that new high-risk area. Dean and Elian [5] found that immigrants to England from Asia and the Caribbean had an increased risk for MS if they moved to England prior to 15 years of age. This finding adds strength to the argument that some environmental variable must have a role to play in the development of MS.

In 1877, Charcot was the first to publish a paper that recognized that the prevalence of MS was not uniform. He discovered that, at the time, there appeared to be a higher prevalence of MS in France compared with Germany or England [6]. Since that time, numerous prevalence studies have been published which suggest that a north-south gradient of MS prevalence appears to exist in the northern hemisphere. While there is evidence to suggest that this gradient is disappearing in some countries [7], a recent meta-analysis by Simpson Jr. et al. [8] shows that on a global scale, the latitudinal gradient still exists with exception being in Italy and northern Scandinavia.

MS is generally most prevalent in northern geographic latitudes. The highest rates of MS prevalence are generally found between the latitudes of 45 degrees north and 65 degrees north [9]. This same latitudinal prevalence rate can be found in similar latitudes in the southern hemisphere. The disease is very rare near the equator.

## 2. Global Prevalence Zones

Kurtzke [10] designated a three-zone global prevalence rating: high zones (30–80 per 100,000), medium zones (5–25 per 100,000), and low zones (<5 per 100,000). The high zones for MS prevalence are generally found in Canada, Northern United States, most of Northern Europe, New Zealand, Australia (south eastern), and Israel. Medium Zones included southern Europe, southern United States, and northern Australia. Low zones included Asia, most of Africa, and South America. Rosati [11] published a paper which attempted to update the global prevalence of MS in the world. Rosati's most notable findings were that there are “many exceptions to the previously described north-south gradient” and that MS is very rare among certain races such as Samis, Turkmen, Uzbeks, Kazakhs, Kyrygzs, native Siberians, native North and South Americans, Maoris, African blacks, Chinese, Japanese, and Canadian Hutterites.

The high, medium, and low zones described by Kurtzke are still valid but a significant number of MS prevalence studies demonstrating rates well above 100 per 100,000 (12–35) may suggest that the scale is not accurately capturing the designated high zones of MS.

## 3. Method

The search terms “multiple sclerosis and prevalence” and “multiple sclerosis and epidemiology” were applied to the following data bases:

- Medline—1950-present
- Medline—In-process and other non-indexed citations
- CINAHL—1982-present
- HealthSTAR—1966-present
- Health and Psychosocial instruments 1985–2007.

Where possible studies which used McDonald or Poser criteria were selected. 57% of the prevalence studies used either Poser or McDonald criteria. The remainder of the studies used population surveys, older diagnostic criteria such as Bauer, Rose, Allison and Millar, and Schumacher, or unknown diagnostic criteria. With the above filters (including English language studies only) and search criteria applied, we found 131 prevalence data sets (geocases) that were analyzed with descriptive statistics spatial statistical analysis tools.  Table 1 shows a sample of the MS geocases and the Case Ascertainment Methods and Criteria for Diagnosis.

Table 2 shows a sample of the area studied in the MS prevalence studies as well as the latitude, longitude, and prevalence rates per 100,000. The sample data set table provides an example of the center-point latitude and longitude used for the relevant studies. If the study population is defined by a city, then the latitude and longitude are those generally accepted for the city to the nearest minute. When larger regions were studied such as at a province, state, region or country level, and a city-level, center-point for the area will be calculated and the latitude and longitude of that center-point to the nearest minute will be used. The areas in parentheses are the approximate center-point of the area.

Spatial statistical analyses was completed using the ESRI ArcGIS-ArcInfo [12] spatial statistics toolbox [13]. The specific analyses contained in each category that were used in the data analysis are listed below.

- Geographic Distribution
  - Median Center
  - Mean Center
  - Central Feature
  - Linear Directional Mean
  - Directional Distribution (Standard Deviational Ellipse)
  - Standard Distance.
- Pattern Analysis
  - Average Nearest Neighbor
  - High/Low Clustering (Getis-Ord General *G*)
  - Spatial Autocorrelation (Morans *I*)
  - Multi-Distance Spatial Cluster Analysis (Ripley's *k*-function).
- Cluster Mapping
  - Cluster and Outlier Analysis (Anselin Local Moran's *I*)
  - Cluster/Outlier Analysis with Rendering
  - Hot Spot Analysis (Getis-Ord Gi*)
  - Hot Spot Analysis with Rendering.

## 4. Results

Using the search criteria outlined in Methods section, we found 131 geocases that met the criteria.  Table 3 shows the descriptive statistics containing the 131 geocases. Of note is the mean global prevalence rate of MS at 67.83 per 100,000 and the maximum value of 350 per 100,000 (Canada).

## 5. Spatial Statistics

**Figure 1:**
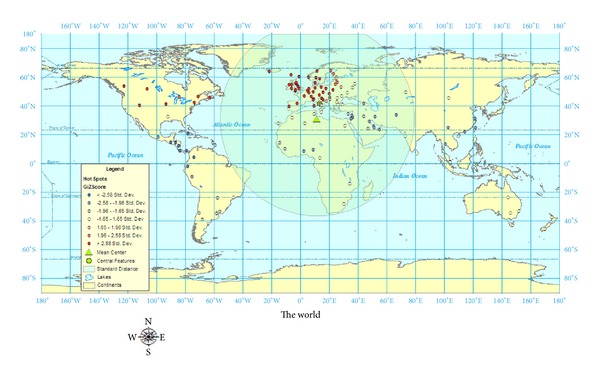
World map of MS prevalence hot spots, Mean Center, Central Feature, and Standard Distance using 131 geocases.

**Figure 2:**
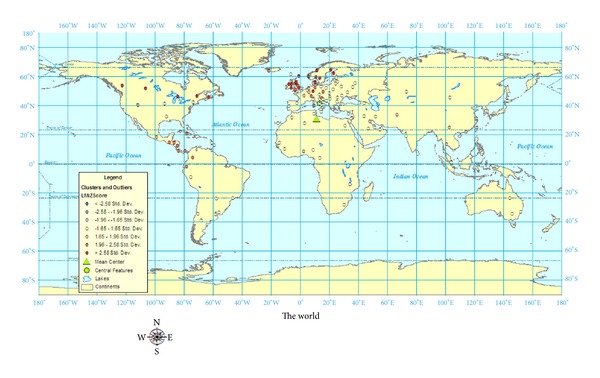
World map of MS prevalence analysis of clusters and outliers using 131 geocases.

To examine the data from the 131 geocases visually, Arc GIS 9.3.1 was used to generate maps on a global level. What is immediately evident from the maps in Figures 1 and 2 is that the majority of the geo-cases represent prevalence studies from Europe. We can see, in Figure 1, Hot Spots, Mean Center, Central Feature, and the Standard Distance of the 131 geo-cases for MS prevalence in the world. The Mean Center of the 131 geo-cases appears to be in northern Africa at approximately 30°, 22′ N and 11°, 4′ E. This center merely represents the Mean Center location of the average of the *x* and *y* values for the geo-cases and should not be confused with a geographic center for MS prevalence. This information tells us that the majority of studies used in this research were based on research from the northern hemisphere. Similar to Mean Center, Central Feature represents a point that is the smallest distance to all the other points. The Central Feature in Figure 1 is located in approximately the middle of Italy at 42°, 0′ N and 13°, 1′ E. The Standard Distance which is the transparent circle encircling all of Europe and Africa again demonstrates that the majority of the geo-cases are representative of data from European countries.

The Gi statistic (hot spot analysis) examines whether features with high or low values tend to cluster in an area. This statistic tool will examine whether neighbouring points have similar values; if they are similar, then they are considered hot spots. A *Z* score is used to determine the statistical significance of the relationship between the neighbours. A high positive *Z* score as seen in most of Europe and Canada represents a *Z* score which is more than 2.58 SD from the mean. The areas that are less than −2.58 SD represent areas of low prevalence of MS. 

Figure 2 shows a spatial analysis done to examine for clusters and outliers. Using the Cluster and Outlier Analysis (Anselin Local Morans *I*) tool, the data is analyzed for values of similar magnitude and those which would be very dissimilar (outliers). This index (Local Morans *I*) then has a *Z* score calculated to determine the significance. The high, positive *Z* scores (red dots) represent clusters which have similar values to values to surrounding points. A low, negative *Z* score (yellow dots) represents an outlier which means that the values are significantly (*P* = 0.05) different from surrounding values. The significant clusters are in the areas of Canada, United Kingdom, and Scandinavia. The significant outliers are found in Eastern Europe, South America, and Africa.

**Figure 3:**
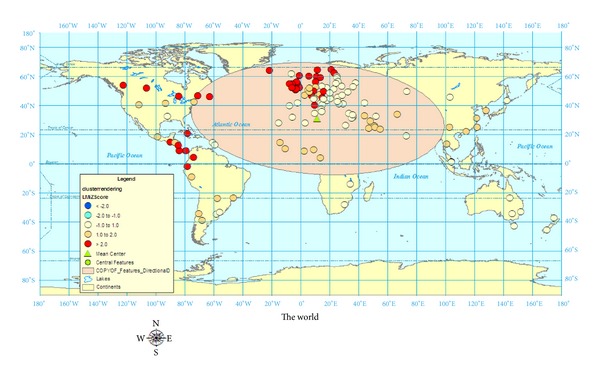
World map of MS prevalence with cluster rendering and directional distribution using 131 geocases.

Figure 3 shows cluster analysis with rendering and a directional distribution of MS prevalence. In addition to seeing the clusters with color-weighting, the directional distribution (Standard Deviational Ellipse) shows where the majority of the geocases are in Europe and trending towards Canada and south-east Asia.

## 6. Pattern Analysis

Using ArcGIS 9.3, a statistical test of pattern analysis was performed to quantify the relationships of the geocases. While mapping the geocases, as shown in Figures 1–3, gives a sense of patterns, statistically testing using pattern analysis quantifies the probabilities that the visual relationships did not merely occur by chance. The four pattern analyses performed on the 131 geocases were Average Nearest Neighbour Distance, High/Low Clustering, Spatial Autocorrelation, and Multidistance Spatial Cluster Analysis.

Average Nearest Neighbour: this statistical tool measures the distance between the centroid of a geocase and its nearest neighbour's geocase centroid. These distances are then averaged to determine if a cluster exists relative to a random distribution. An index ratio is created (observed distance divided by expected distance). An index less than 1 is trending towards a cluster whereas an index greater than 1 is trending towards dispersion. The Average Nearest Neighbour test results with our 131 geocases revealed that significant clustering exists: 0.67 (index), *Z* = −7.29 SD, *P* < 0.01.

High/Low Clustering (Getis-Ord General *G*): this test determines how concentrated high or low the geocase values are in the area of our global map. The results of this statistical tool revealed significant high clusters: General *G* index = 0.04, *Z* = 7.16 SD, *P* < 0.01. The input variable used for this test was MS prevalence.

Spatial Autocorrelation: Morans *I*: this test will determine how similar the geocases are based on both their location and their values (MS prevalence). As with the Getis-Ord General *G*, it uses an index to determine the degree of clustering. As with the previous 2 tests, a score and a *P* value are calculated. A Moran's *I* index near 1.0 trends to clustering whereas −1.0 trends towards dispersion. The results of this test for the 131 geocases demonstrate significant clustering: Moran's *I* index = 0.43, *Z* = 13.42 SD, *P* < 0.01.

Multidistance Spatial Cluster Analysis: Ripley's *k*-Function: this tool as with the previous two tools measures the degree of clustering. Unlike the previous two tools, this tool analyzes clusters over a range of distances. For the test of the 131 geocases, we chose 10 distance bands over which to have the tool analyze clusters in each of the 10 distance bands. If the observed number of clusters is higher than the expected number of clusters, then the test is significant for clustering in that band. The index used is the Ripley's *k*-Function and is expressed as *L* (*d*). The higher (and more positive) the Ripley's *k*-Function means that the geocases are more clustered. The results of this test reveal that all distance intervals from 7.44 to 74.44 are significantly clustered except at 74.44 which is slightly dispersed: *L*(*d*) = 72.04, diff = −2.40. The most clustered distance was at 29.78: *L*(*d*) = 51.28, diff = 21.50.

## 7. Discussion

Although Kurtzke did revisit the global prevalence scale for MS when he reexamined more recent European data, the original, three-zone scale of High, Medium, and Low remains the same. When our 131 prevalence studies were examined, over forty percent of the prevalence rates were over 50 per 100,000. It is clear that Kurtzke's [10] original divisions of MS prevalence into high zones (30–80 per 100,000), medium Zones (5–25 per 100,000), and low zones (<5 per 100,000) may not be granular enough to account for more recent prevalence studies showing MS prevalence rates in Canada over 300 per 100,000. As a result of this research, it is recommended that the global prevalence scale be expanded into a five-zone scale based on the results from the spatial analyses in Figures 1–3. The prevalence scales generated by ArcGIS divided the five prevalence zones into (rounded): 0–13; 13–38; 38–70; 70–170; 170–350 per 100,000. The categories representing these more granular divisions for global MS prevalence zones should be very high (170–350 per 100,000), high (70–170 per 100,000), medium (38–70 per 100,000), low (13–38 per 100,000), and very low (0–13 per 100,000).

It is necessary to expand from a three-zone classification system to a five-zone classification system to avoid grouping together countries with prevalence rates which are incomparable. In a three-zone classification, countries such as England, Germany, and Switzerland which have prevalence rates between 100 and 150 per 100,000 would likely be ranked as “high” and in the same category as countries such as Canada, Norway, Sweden, Scotland, and Ireland which have prevalence rates ranging between 150 and 350 per 100,000. A similar argument is made for adding a “very low” category as to not group countries such as Malaysia, Thailand, and most of the African countries with a zero to very low prevalence rates with countries such as Argentina, Uruguay, and the north island of New Zealand which have prevalence rates between 13 and 38 per 100,000.

Whether due to actual increases in MS incidence rates or better diagnostic practices in some countries, it is likely that in the future, the proposed five-zone classification scale for global MS prevalence will be even more important to capture and effectively compare countries with higher MS prevalence rates than previously reported.  Table 4 compares Kurtzke's [10] global MS prevalence scale and the new, Proposed global MS prevalence scale.

## Figures and Tables

**Table 1:** Sample table of MS prevalence secondary sources and case ascertainment methods.

Region and journal reference	Prevalence data (per 100,000)	Prevalence year	Criteria for diagnosis	Case ascertainment method(s)
Canada [14]	Atlantic Region: 350 Quebec: 180 Ontario: 230 Prairie Region: 340 British Columbia: 240	2000-2001	Self-report	Canadian Community Health Survey 1.1

United States [15]	Western Region: 97.5 Midwestern Region: 96.0 Northeastern Region: 96.5 Southern Region: 65.5	1982–1996	Self-report	National Health Interview Survey

Russia [16]	35–70	2000	Unknown	Multiple Sources

**Table 2:** Sample dataset table of latitude and longitude and prevalence.

Country and region/city (any city in parentheses is an approximate regional center)	Latitude and longitude	MS prevalence rate (per 100,000)
Canada (Beck et al., 2005 [14])		
Atlantic Provinces (Charlottetown)	46°14′N 63°7′W	350
Quebec (Quebec city)	46°48′N 71°14′W	180
Ontario (Sault Ste. Marie)	46°30′N 84°20′W	230
Prairie Provinces (Saskatoon)	52°6′N 106°37′W	340
British Columbia (Prince George)	53°55′N 122°50′W	240
United States (Noonan et al., 2002 [15])		
Western Region (Salt Lake City)	40°41′N 111°53′W	97.5
Midwestern Region (Des Moines)	41°35′N 93°37′W	96.0
Northeastern Region (Albany)	42°39′N 73°45′W	96.5
Southern Region (Little Rock)	34°45′N 92°18′W	63.5
Russia (Gusev et al., 2002 [16])		
Moscow	55°45′N 37°42′E	40.8
Orel	52°59′N 36°5′E	64.8

**Table 3:** Descriptive statistics for 131 geocases of MS prevalence data.

	MS prevalence (per 100,000)
*N *	
Valid	131
Missing	0
Mean	67.8318
Std. error of mean	6.18799
Median	42.8700
Std. deviation	70.82478
Minimum	0.00
Maximum	350.00

**Table:** (a) Kurtzke's global MS prevalence scale

Classification	Prevalence rate (per 100,000)	Countries
High	30–80	Most of Europe, most of United States, Canada, New Zealand, Israel, Cyprus, and south-eastern Australia

Medium	5–25	Southern Europe, most of Australia, most of Russia from the Urals into Siberia as well as the Ukraine, South Africa, possibly much of the Caribbean region, and South America

Low	<5	Asia, most of Africa, and South America (Venezuela and Colombia)

**Table:** (b) Proposed global MS prevalence scale

Classification	Prevalence rate (per 100,000)	Countries
Very high	170–350	Canada, Sweden (Varmland), Finland (Seinäjoki and Vaasa), Scotland, and most of Ireland

High	70–170	Most of United States, Norway, Sweden (Västerbotten), Denmark, Finland (Uusimaa), Iceland, England, Ireland (Wexford), Germany, Austria, Switzerland, Belgium, Italy, Turkey, Slovenia, Croatia, Czech Republic, Luxembourg, and Netherlands

Medium	38–70	Southern United States, Russia, most Australia, New Zealand (south), Faroe Islands, Poland, Estonia, Spain, Greece, Hungary, Serbia and Montenegro, Bulgaria, Belarus, Israel, Latvia, Lithuania, Moldova, Portugal, and Ukraine

Low	13–38	Australia (Queensland), New Zealand (north), Kazakhstan, Romania, India, Jordan, Saudi Arabia, Martinique, Argentina, South Africa, Brazil, Bahrain, Barbados, Lebanon, Malta, Morocco, Slovakia, Tunisia, United Arab Emirates, and Uruguay

Very low	0–13	Japan, Mexico, China, Taiwan, Malaysia, Thailand, Kuwait, Panama, Colombia, Afghanistan, Algeria, Bahrain, Cameroon, Chile, Costa Rica, Cuba, Ecuador, Guatemala, Guinea, Honduras, Iraq, Malawi, Mongolia, Nicaragua, Nigeria, Paraguay, Peru, Qatar, Republic of Korea, Singapore, Benin, and Senegal
